# Genome Sequence of the Acetogenic Bacterium *Butyribacterium methylotrophicum* DSM 3468^T^

**DOI:** 10.1128/genomeA.01338-16

**Published:** 2016-12-01

**Authors:** Frank R. Bengelsdorf, Anja Poehlein, Bettina Schiel-Bengelsdorf, Rolf Daniel, Peter Dürre

**Affiliations:** a Institut für Mikrobiologie und Biotechnologie, Universität Ulm, Ulm, Germany; b Genomic and Applied Microbiology and Göttingen Genomics Laboratory, Georg-August University Göttingen, Göttingen, Germany

## Abstract

*Butyribacterium methylotrophicum* DSM 3468^T^ is an acetogenic methylotrophic, anaerobic, carbon monoxide–oxidizing bacterium that produces acetate, butyrate, and butanol. The genome consists of a single chromosome (4.3 Mb) and harbors 3,989 predicted protein-encoding genes.

## GENOME ANNOUNCEMENT

*Butyribacterium methylotrophicum* DSM 3468^T^ is a Gram-positive, motile bacterium that was isolated from a sewage digester in Marburg (Gemany) by Zeikus et al. in 1980 (1). This autotrophic acetogen can reduce H_2_ + CO_2_ and CO using the Wood-Ljungdahl pathway and produces acetate, butyrate, and butanol (2). In general, the metabolic features of *B. methylotrophicum* appear to be similar to those of *Eubacterium limosum* ATCC 8486^T^ (3), and phylogenetic analysis of the 16S rRNA gene sequences also indicated a close relationship between both strains (4, 5). *B. methylotrophicum* and related strains harbor metabolic characteristics, which make these organisms ideal candidates for a syngas fermentation process (6), although *B. methylotrophicum* was provisionally classified as a risk level 2 organism by the Deutsche Sammlung von Mikroorganismen und Zellkulturen.

Extracted DNA was used to generate Illumina shotgun paired-end sequencing libraries, which were sequenced with a MiSeq instrument and the MiSeq reagent kit version 3, as recommended by the manufacturer (Illumina, San Diego, CA, USA). Quality filtering using Trimmomatic version 0.32 (7) resulted in 4,256,529 paired-end reads with an average read length of 301 bp. The assembly was performed with the SPAdes genome assembler software version 3.5.0 (8). The assembly resulted in 27 contigs (>500 bp) and an average coverage of 94-fold. The assembly was validated and the read coverage determined with QualiMap version 2.1 (9). The draft genome of *B. methylotrophicum* DSM 3468 consists of a single chromosome (4.3 Mb) with an overall GC content of 47.5%. Automatic gene prediction and identification of rRNA and tRNA genes was performed using the software tool Prokka (10). The draft genome contained nine rRNA genes, 55 tRNA genes, 2,890 protein-encoding genes with function prediction, and 1,099 genes coding for hypothetical proteins.

Genes encoding enzymes of the methyl and carbonyl branches of the Wood-Ljungdahl pathway are conserved within acetogenic bacteria (11). Correspondingly, the genome of *B. methylotrophicum* harbors a gene cluster that is highly similar to the respective cluster of *E. limosum* ATCC 8486^T^ and harbors genes encoding the Rnf complex. A comparative genome analysis of the strains *E. limosum* ATCC 8486^T^ (12) and *E. limosum* KIST612 (13) with *B. methylotrophicum* DSM 3468 revealed high average nucleotide identities of 94.6% and 99.7%, respectively. The threshold range for species differentiation is 95 to 96% (14). A further comparison revealed that the genome of strain DSM 3468 (4,256,529 bp) is smaller than that of ATCC 8486^T^ (4,370,113 bp) and of similar size to KIST612 (4,276,902 bp), indicating that *B. methylotrophicum* is a distinct species compared to *E. limosum* ATCC 8486^T^.

### Accession number(s).

This whole-genome shotgun project has been deposited at DDBJ/ENA/GenBank under the accession number MIMZ00000000. The version described in this paper is the first version, MIMZ01000000.
